# Graph-based representations in modern protein science

**DOI:** 10.1042/BCJ20260179

**Published:** 2026-09-04

**Authors:** Dana S. Matthews, Sacha B. Pulsford, Anthony Barancewicz, John Z. Chen, Barnabas Gall, Mahakaran Sandhu, Matthew A. Spence, Colin J. Jackson

**Affiliations:** 1 Research School of Chemistry, The Australian National University, Canberra, ACT 2601, Australia; 2 ARC Centre of Excellence for Innovations in Peptide & Protein Science, Research School of Chemistry, The Australian National University, Canberra, ACT 2601, Australia; 3 Research School of Biology, The Australian National University, Canberra, ACT 2601, Australia; 4 ARC Centre of Excellence in Synthetic Biology, Research School of Biology, The Australian National University, Canberra, ACT 2601, Australia; 5 ARC Centre of Excellence in Mathematical Analysis of Cellular Systems, Research School of Biology, The Australian National University, Canberra, ACT 2601, Australia

**Keywords:** protein evolution, protein structure, protein-protein interactions

## Abstract

For more than 50 years, the linear sequence and the multiple sequence alignment have been the foundational data structures of protein science, and they remain central to homology search, phylogenetic inference, covariance-based contact prediction, and modern protein language models. However, relational and graph-based representations are increasingly being adopted alongside sequence-based methods to capture biological relationships that linear data structures express only implicitly. Proteins fold as three-dimensional residue interaction networks, evolve through high-dimensional genotype networks defined by mutational connectivity, and operate within cellular protein–protein interaction graphs. Here, we review how graph theory is being used to describe and understand these relationships across protein science, with an emphasis on what these methods offer biochemists working on enzyme superfamilies, protein engineering, drug targets, and functional annotation. We trace the development of these ideas from early theoretical topologies, through statistical coupling and the structural network analyses, to the geometric and graph-like representations used in recent machine-learning-driven advances. Throughout, we emphasise that graphs do not replace sequences or MSAs but provide a complementary representation for biochemical relationships that are difficult to express in one dimension.

## The relational nature of protein science

A graph, in its simplest form, is a set of nodes connected by edges, where each edge records that two things are related (e.g., two residues interact, two sequences differ by one mutation, two proteins bind, etc.). It describes a system by these connections, i.e., by what is linked and how. Reducing a system to a single kind of relationship in this way is referred to as an abstraction. In the present review, we describe different graphical abstractions often used to describe biological systems and what the connectivity within these different representations can tell us.

Biology is shaped by webs of connectivity, with function arising from the discrete and relational nature of components. For example, at the molecular scale, a folded protein consists of residues from the 20 canonical amino acids, and its three-dimensional structure (and therefore its function) is defined by how those residues interact in space and in time (dynamics). At the cellular scale, proteins themselves can be represented as nodes, with edges connecting any two proteins that physically interact. Both are descriptions of a graph, even though their nodes and edges denote very different things. Once nodes and edges are defined, edges can carry weights (e.g., the strength of an interaction or the distance between residues) or direction (e.g., an evolutionary trajectory from one genotype to a fitter one), and graphs can be layered (a residue contact graph coupled to a co-evolution graph). It is this flexibility that makes graphs a useful common language across biological scales.

**Figure 1:**
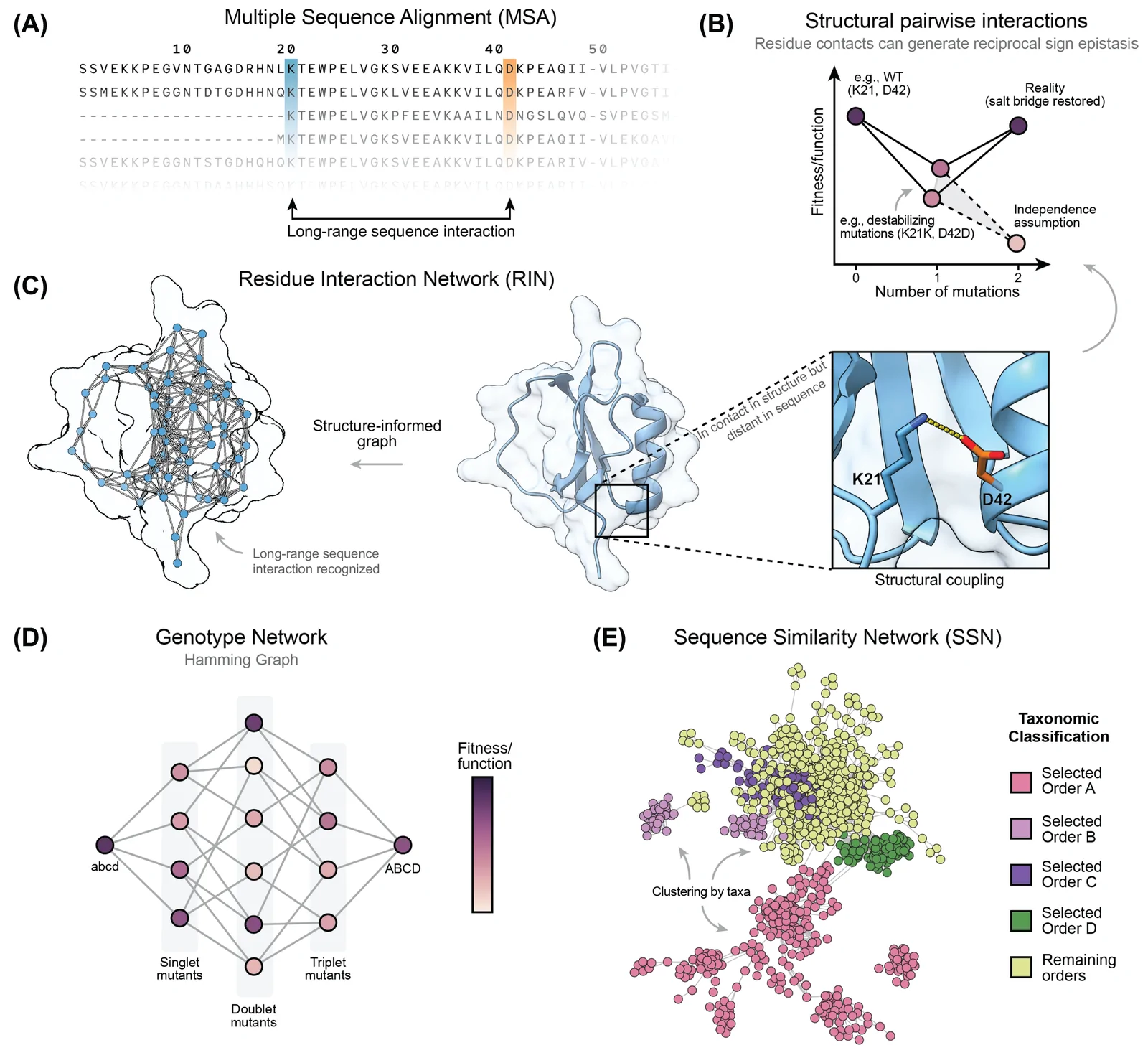
Graph representations of protein sequence and structure (**A**) An example multiple sequence alignment (MSA) represents homologous protein sequences as rows with conserved sites aligned in corresponding columns. Two sites are highlighted in blue and orange that interact in the three-dimensional structure but appear distal when the sequences are visualised as a linear string. (**B**) Despite being far away in an MSA, sites can be close in structure (or interact indirectly), resulting in epistasis and breaking the assumption of MSAs that residues are independent. (**C**) RINs are contact graphs where the three-dimensional fold is used to make a structure-informed graph. In this graph, residues that interact in the structure are captured in the underlying graph. (**D**) Within a genotype network (Hamming graph), nodes represent sequences and edges connect sequences differing by one mutation. Selection samples only a sparse subset of this combinatorial Hamming graph, producing (**E**) an observed SSN, in which nodes correspond to sequences or sequence clusters present in the alignment and edges represent sequence similarity.

In a graph-theoretic framework, a protein is a relational object, though what it corresponds to depends on the scale. At the atomic level, the protein itself is a network: a residue interaction network (RIN) where nodes are amino acids and edges are physical contacts [1]. At the evolutionary level, the protein is instead a single node in a genotype network connected to other sequences by single mutational edges [2] (Figure 1D). At the systems level, it is again a single node, this time in a sparse sequence similarity network (SSN) made up of the sequences that selection has retained [3] (Figure 1E). At the functional level, it is one node in a larger interaction graph, such as a protein–protein interaction (PPI) network [4]. Describing a protein this way allows problems to be reframed as operations on a graph: allostery can be analysed through centrality (how much of the network’s connectivity sits on a residue) and path length (how many connections exist between one residue and another) in a residue graph [5–7], evolvability as the accessibility of adaptive walks (stepwise walks through sequence space that improve fitness one mutation at a time) across a genotype graph [8–10], and substrate specificity across an enzyme superfamily as the way an SSN partitions into functional clusters, where groups of sequences are related to one another more than the rest of the network [3,11,12].

To date, methods to study protein evolution have largely been organised by a single, alternate geometric concept: alignments. Since the formulation of the Central Dogma [13], the linear sequence has been regarded as the central data structure for biological information, and the MSA as the primary analytical tool (Figure 1A). Consequently, databases are often organised as rows of strings, and evolutionary history is inferred by computing edit distances between them [14]. This linear view has been enormously productive. It enabled Dayhoff to compile the first Atlas of Protein Sequence and Structure and formulate the PAM (point accepted mutation) matrices [15], and it underpinned the development of BLAST (basic local alignment search tool) [16] and the annotation of the first genomes. The MSA itself remains integral to bioinformatics: it is a key input to AlphaFold’s Evoformer [17], the basis for covariance-based contact prediction [18], and a benchmark against which protein language models are evaluated [19]. Nevertheless, constraining analyses to a single underlying approach risks limiting or even biasing conclusions/findings, offering at best a partial view of the system and processes at play.

In the case of MSAs, one such limitation is site independence. Many classical analyses built on MSAs treat alignment columns independently: under standard substitution models, the probability of a residue at position *i* is modelled as conditionally independent of position *j* [20], and pairwise tools such as BLAST and standard phylogenetic likelihood methods, which treat each site as evolving independently, inherit this assumption [16,20]. This site independence can miss coupling between residues that sit far apart in the sequence but interact in the folded structure, where a change at one position is only tolerated alongside a compensating change at the other (Figure 1B). Modern analyses of MSAs analyse pairs of positions together, both within a protein [18,21] and between interacting proteins [22], to overcome this to some extent. Direct coupling analysis [18], Potts coupling methods such as plmc [21], the MSA Transformer [23], and AlphaFold’s Evoformer [17] all exploit pairwise statistics in alignment columns to recover contacts and improve folding accuracy. These methods represent a shift from the site-independent treatment of residues to representations and analyses that explicitly capture pairwise and higher-order relationships, which better reflect the physical basis of protein behaviours. For example, a mutation at one site may destabilise the structure unless compensated at a distant site hundreds of residues apart [24] (Figure 1B); these residues would appear unrelated in linear sequence but would be revealed as neighbours in a contact graph (Figure 1C). Furthermore, while native structure is determined by the amino acid sequence [25], the folding pathway from linear primary sequence to tertiary structure is a cooperative collapse formed through the formation of local contact networks [26].

This does not displace linear representations. Alignments are a key component of many structure prediction models [17], and sequence models can recover contacts from alignment statistics [23]. But sequence alone does not state the relationships between residues, which must be inferred anew for each question asked. Indeed, models supplied with structural and sequence information outperform those supplied with sequences alone [27–29]. Where one-dimensional representations obscure relationships, graphs and other relational representations that capture connectivity, clustering, and topology declare them directly.

The value of graph theory in protein science lies in its ability to express and unify biological relationships that are difficult to represent in a single linear sequence. Deploying alternative, complementary graph representations can capture different types and levels of relationships that often determine biological behaviour, including residue contacts, evolutionary trajectories, and functional interactions. Our goal in the present review is to highlight when graph representations clarify familiar problems to facilitate their effective use in the literature more broadly, with a specific focus on their use in elucidating aspects of molecular evolution/sequence-space analyses, for example: (i) functional annotation, or how enzyme superfamilies partition into functional groups; (ii) how active-site and allosteric residues are connected in folded structures; (iii) how mutational paths are blocked by epistasis; and (iv) why modern protein-design models treat proteins as relational objects. Each section takes a class of biological question, explains its underlying graph, and highlights what that representation enables.

## A taxonomy of protein graphs

It is useful to define the various graphs used to study biological systems because the appropriate analyses, software, and biological caveats differ. Table 1 summarises the major classes described in the present review. These graph classes are linked; e.g., an SSN can be used to identify sequences to include in an analysis of a genotype network [30–32]. The classes nonetheless answer different questions and differ in what they represent. Some pose biological hypotheses about physical or evolutionary connectivity that can subsequently be falsified by experiment. In contrast, in a graph neural network (GNN) the nodes and edges are picked to give the network a useful structure to learn from. ProteinMPNN [33], for example, connects each residue to its nearest neighbours in space, so the model can reason over local structure. The two approaches can look alike because both represent a system as nodes and edges, but an edge means something different in each, and reading a model’s graph as if it relates to biological functions is a common source of overinterpretation.

**Table 1:** The major classes of graph used in protein science

Graph	Nodes	Edges	Biological question	Suitable data	Approaches	Potential misuse
Genotype network	Single protein sequences	Single amino acid substitutions between sequences	How are functional sequences distributed in sequence space, and can evolution move between them?	Dense samplings of a single protein/scaffold, ideally with intermediate variants present	Custom DMS analyses; NetworkX [34]	Treating an SSN as a genotype network: SSN edges span many substitutions, not single steps.
SSN	Protein sequences	Pairwise similarity above a chosen threshold (e.g., BLAST bitscore [16]; MMseqs2 [35])	Functional partitioning of large superfamilies; identification of dark sequence space.	Large sets of homologous proteins with detectable similarity	EFI-EST [36], Cytoscape [37]; ProteinCluster Tools [12]	Reading cluster boundaries, which can shift with the chosen similarity threshold, as fixed functional divisions; treating edges as evolutionary relationships when they encode only similarity.
Phylogenetic trees	Existing protein sequences (tips), statistically inferred ancestral sequences (internal nodes)	Evolutionary distance (substitutions per site) between ancestor and descendant.	What is the evolutionary history of a set of proteins? Assumes that alignment columns evolve independently and evolution is bifurcating.	Aligned homologous sequences with sufficient variation, the alignment itself should be reliable	IQ-TREE [38,39]; BEAST [40]	Forcing a bifurcating tree onto reticulate histories shaped by recombination or horizontal transfer; treating inferred ancestral sequences as if experimentally validated.
RIN	Amino acid residues within a single protein	Non-covalent contacts or Cα/Cβ proximity within a cutoff (e.g., 5–8 Å)	Allosteric pathways, folding nuclei, identification of catalytic and hot-spot residues.	Resolved or high-confidence predictions of protein structures	RINalyzer [36]; RING [41]; NAPS [42]	Reading high centrality scores as proof of mechanism without dynamics, ensembles or experimental confirmation.
PPI network	Proteins (or genes)	Physical or genetic interactions, often from high-throughput screens	Cellular organisation, modular function, disease modules, drug target prioritisation.	Confidence-scored interactions from a defined organism, cell type, or condition	STRING [43]; BioGRID [44]; IntAct [45]	Treating Y2H, AP-MS, and co-citation edges as equally reliable; ignoring study bias and edge confidence.
Knowledge graph	Heterogeneous entities (proteins, structures, GO terms, taxa, drugs, phenotypes)	Typed relationships (interacts with, found in, catalyses)	Function inference for orphan proteins by triangulation across entity types.	Heterogenous datasets linked by typed relationships	UniProt RDF [43]; Reactome [46]; GO-CAM [47]; KG-Hub [48]; STRING [43]	Inferring new annotations from paths that themselves rest on inferred annotations, producing circular evidence, producing circular evidence chains.
Geometric structure (GNN) graph in deep learning	Residues or atoms (structure GNNs); any of the above for other tasks.	Geometric proximity, with rich edge features encoding distance and orientation	Can structure and geometry be built directly into a model for prediction and design?	Any graph structure that is suitable as input into a neural network for training and prediction	General frameworks: PyTorch Geometric [49], Deep Graph Library [50]; Protein models: ProteinMPNN [33]; RFdiffusion [51]	Reporting prediction accuracy alone as evidence of mechanistic understanding; ignoring training-data bias and over-smoothing.

AP-MS, affinity purification-mass spectrometry; BEAST, Bayesian evolutionary analysis sampling trees; BioGRID, Biological General Repository for Interaction Datasets; BLAST, Basic Local Alignment Search Tool; Cα, alpha carbon; Cβ, beta carbon; DMS, deep mutational scanning; EFI-EST, Enzyme Function Initiative-Enzyme Similarity Tool; GNN, graph neural network; GO, Gene Ontology; GO-CAM, Gene Ontology Causal Activity Model; KG-Hub, Knowledge Graph Hub; MMseqs2, Many-against-Many sequence searching 2; NAPS, Network Analysis of Protein Structures; PPI, protein–protein interaction; ProteinMPNN, protein message-passing neural network; RDF, Resource Description Framework; RFdiffusion, RoseTTAFold diffusion; RIN, residue interaction network; RING, Residue Interaction Network Generator; SSN, sequence similarity network; STRING, Search Tool for the Retrieval of Interacting Genes/Proteins; UniProt, Universal Protein Resource; Y2H, yeast two-hybrid.

## Understanding the topology of genotype space

**Figure 2:**
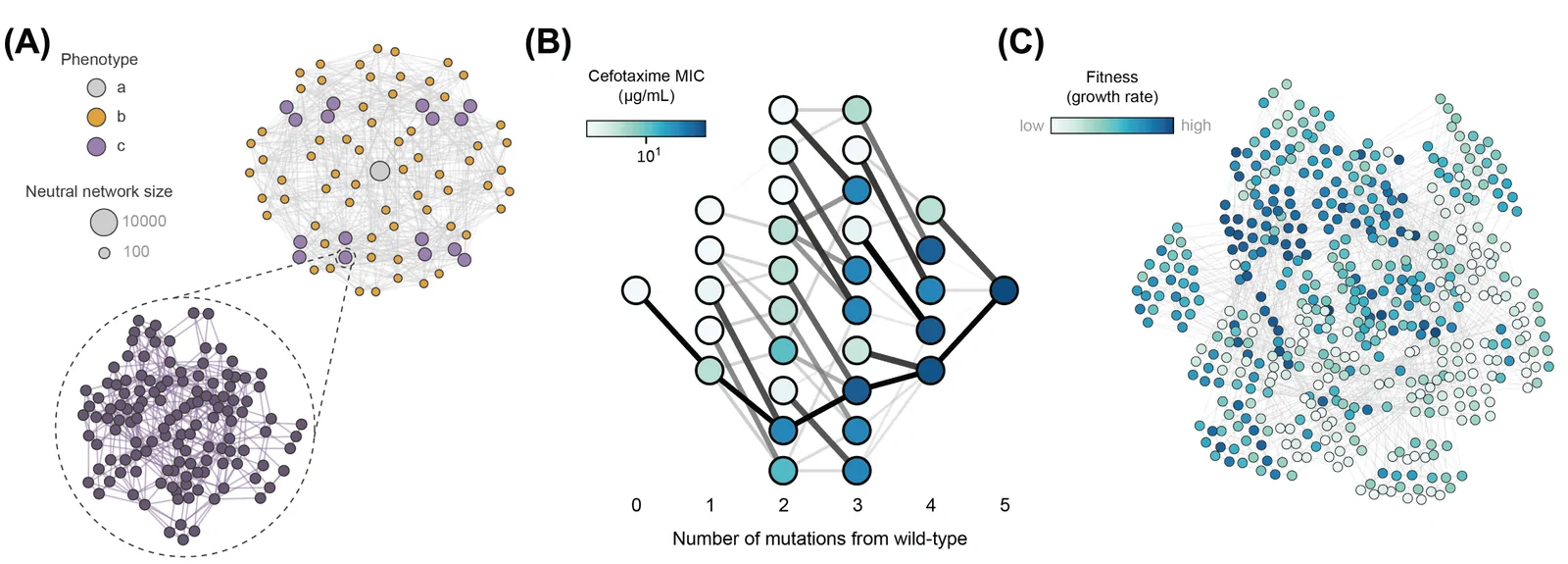
Genotype networks and neutral network connectivity (**A**) A theoretical genotype network showing the relationship between variants with phenotypes a (grey nodes), b (yellow nodes) and c (purple nodes). A view of a collapsed neutral network of a purple network is shown. Nodes in this subgraph can be navigated without fitness consequences. This neutral network can be collapsed into a single node in a coarse-grained genotype graph. The landscape model is derived from the polyomino model [54]; evolvability and robustness are computed according to [55]. (**B**) A partial genotype graph of the TEM-1 β-lactamase fitness landscape [8]. Here, node colour denotes the capacity for antibiotic resistance (reported as the minimum inhibitory concentration for the antibiotic cefotaxime). Edge colour indicates the relative probability of each beneficial mutation. Even with relatively few combinatorial mutations (2^5^), the fitness landscape permits only a small fraction of viable adaptive walks (18/120) to the cefotaxime resistance peak. (**C**) Genotype graph of the *Escherichia coli folA* DHFR [56]; a much larger experimental fitness landscape than panel (A), yet the landscape remains navigable across genotype space. A sampled subset of a single adaptive network is shown for clarity.

To appreciate why graph theory is useful in making sense of protein sequence space and molecular evolutionary trajectories, it is important to highlight the combinatorial space proteins navigate. For a protein of 100 amino acids, sequence space comprises 20^100^ (∼10^130^) possibilities. If functional proteins were scattered randomly throughout this space, evolution would be impossible; a random search would almost never find a functional sequence. The fact that sequences can vary and be robust to mutation, implies that the space can be explored by adaptive and/or neutral walks across genotype space, in which single-mutation descendants remain at least as viable as their ancestors. Indeed, it has been hypothesised that the primary determinant in molecular evolutionary outcomes is not the existence of functional sequences, but whether they can be accessed in sequence space without loss of function [52]. The shape of this apparently navigable space is often conceptualised as the topology of a fitness ‘landscape’, an analogy initially formalised by Wright’s seminal work proposing adaptive landscape structures [53]. This leads to the concept of genotype space, a formalisation of these structures that can be represented as a graph wherein a node represents a variant and an edge represents the potential for transition between two variants [53] (Table 1 and Figure 2). Often these are annotated to denote variants or nodes with different functions, while edges may be drawn with different colours or thicknesses to denote the probability of transition, or other such metrics based on fitness or sequence similarity.

Alongside the considerable work on adaptive landscapes, the theory of neutral evolution has also shaped our modern understanding of molecular adaptation [57,58]. Early foundational work by Kimura, and subsequent work, has shown that many genotypes can produce phenotypes that are equally fit (or unfit) under a specific selection regime. These phenotypically redundant variants typically manifest as accessible, connected subgraphs or regions within the genotype map and are referred to as neutral networks. Early computational analysis implied that neutral networks are ubiquitous in genotype graphs because of folding stability: a sequence becomes unfit when it can no longer fold, and thus networks of neutral mutations that do not destabilise the sequence over a stability threshold exist in biomolecules [52,59–63]. This is particularly true for RNA folding genotype graphs, which tend to form large neutral networks, perforated by unfit “holes” in sequence space [64,65]. Codon degeneracy also produces neutrality at the nucleotide level. Synonymous mutations leave a protein unchanged, yet can alter translation rate, co-translational folding, and expression, and therefore can carry a cost of their own [66,67]. Adaptive walks on these holey landscapes transition between distinct neutral networks, i.e., they spread step-by-step from one viable sequence to a connected neighbour, crossing a sequence space in which neutral subgraphs are sparsely connected to one another but internally dense (Figure 2A). Neutral networks can thus be treated as coarse-grained graph nodes whose size and boundary diversity define the phenotypic neighbourhood available to a genetic background [55]. Inter-component connectivity, i.e., between distinct neutral networks, determines whether those phenotypes are reachable without crossing fitness valleys in biologically realistic genotype–phenotype maps [54]. This graph-centric view of neutral evolution has been supported by numerous recent empirical studies on PPI maps [54], ribozyme experimental fitness landscapes [68], synthetic regulatory networks [69], and the neutral effects of mutations from protein structure prediction [70]. A consequence of this is that populations tend towards the densely connected interior of neutral networks where genotypes carry the most neutral neighbours [71], producing latent mutational robustness [72,73]. In practice, this manifests as protein variants with excess stability that can maintain fold and function in the face of increasing mutational load.

If robustness reflects the density and connectivity of neutral networks, evolvability depends on the new phenotypes that become accessible from sampling those networks [52,53,65,74] (Figure 2B). In populations with access to a dense neutral network and thus high mutational robustness, a greater variety of sequences with equivalent fitness may exist in the population. This cryptic variation often results in more frequent instances of variants with diverse promiscuous side activities while maintaining their functional threshold for the native activity [75–77]. This becomes important during evolutionary trajectories involving function changes and/or adaptation to changing selection regimes. While two variants may appear equivalent under one condition or when performing one reaction, such cryptic, neutral variation may mean one variant has latent capacity for an alternate reaction and thus can quickly adapt to a new selection condition and/or acquire a new function [78]. This contingency, i.e., the sensitivity of an evolutionary trajectory to starting sequence context, remains an area of intense study [79,80].

In protein evolution, the phenomenon where a mutation’s fitness effects depend on genetic background is referred to as epistasis [81,82]. This can be identified by observing that combinations of mutations do not result in a simple additive sum of their individual effects. Early empirical landscapes showed that epistasis results in only a minority of possible adaptive walks through a genotype graph being viable. This has been demonstrated in TEM-1 beta-lactamase [8], isopropylmalate dehydrogenase cofactor use [83], Protein G domain B1 [84], TetR transcription-factor binding-site repression [85], tryptophan synthase [86], CTX-M beta-lactamase [87], and antibody binding [88]. Likewise, evolving a phosphotriesterase to an arylesterase and back showed that epistasis can make evolution phenotypically reversible but genotypically irreversible, leaving functionally identical sequences disconnected in the graph [89]. The same phenomenon recurs in protein engineering, where activity mapped onto a genotype graph of engineered variants traces a constructed adaptive walk [90,91]. Many of these studies reinforce earlier suggestions that neutral evolution can keep high-fitness states reachable in large amino-acid alphabets [84,92–94]. A recent example of this is the complete experimental characterisation of the nine-nucleotide/three-codon *E. coli* dihydrofolate reductase (folA) Hamming graph (Figure 2C). This graph is globally navigable because high-fitness genotypes are connected by many accessible routes, despite hundreds of local fitness peaks that would terminate strictly uphill adaptive walks [56].

## Fitness landscapes on graphs

**Figure 3:**
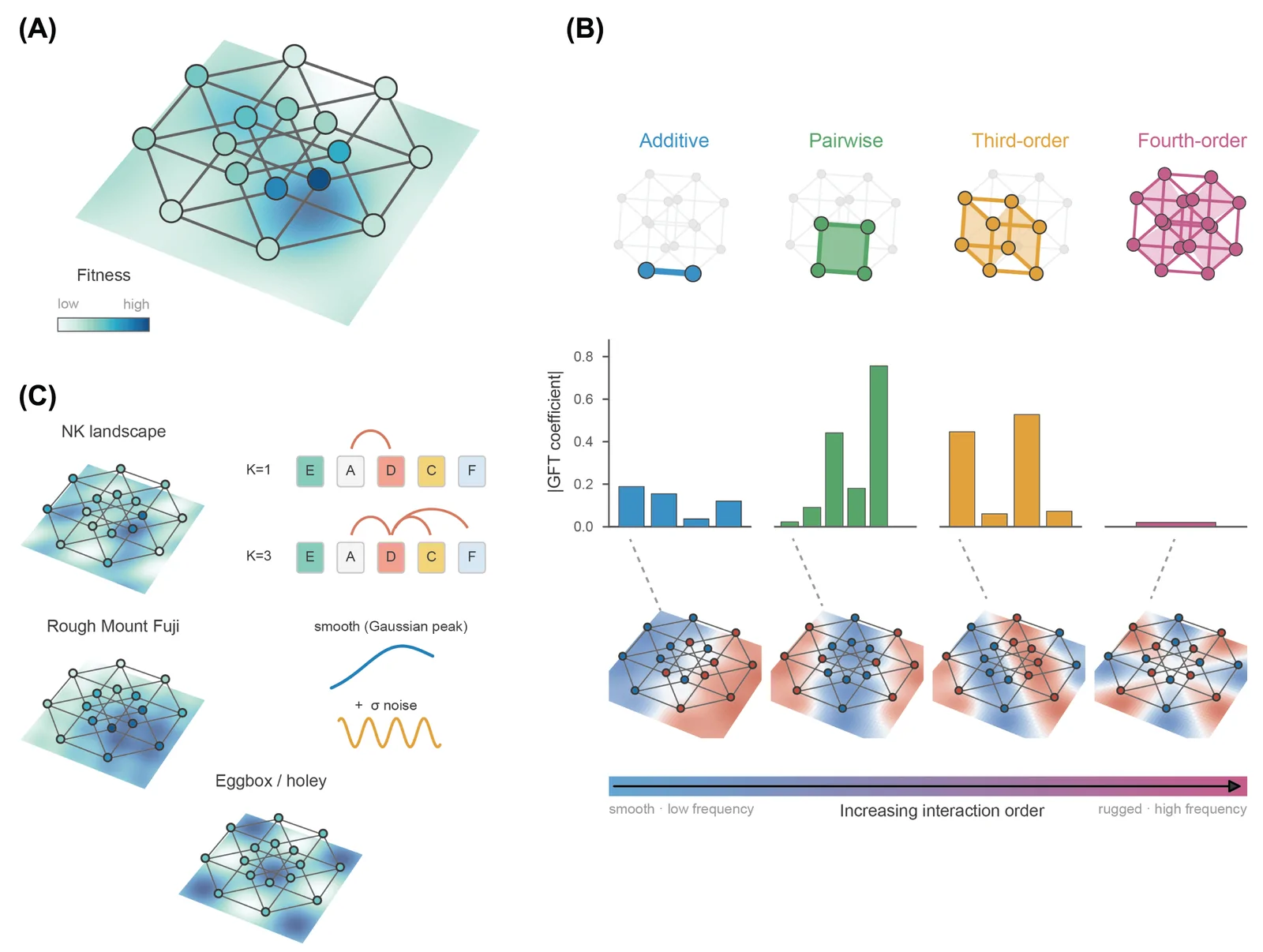
Spectral graph theory of fitness landscapes (**A**) A fitness landscape plots experimental fitness values indexed by a genotype graph, on which accessibility, epistasis, and ruggedness can be measured directly. This is shown as a Hamming graph with nodes (protein sequence variants) coloured by inferred or quantified fitness. The underlying panel displays the continuous fitness inferred from the overlayed discrete space. (**B**) The graph Fourier transform (GFT) decomposes the landscape into components from smooth to rugged; the weight on each shows how much of the fitness variation is smooth versus rugged. On a complete Hamming graph these components are the additive, pairwise, and higher-order mutational effects, giving a quantitative measure of epistasis. (**C**) Synthetic landscape models: NK tunes ruggedness through higher-order interactions, set by *K*; rough Mount Fuji (RMF) adds a small random term to a smooth gradient; eggbox landscapes alternate fit and unfit genotypes.

A genotype graph on its own gives a qualitative picture of evolution and function. However, mapping experimental fitness values onto its nodes turns it into a fitness landscape that can be analysed quantitatively (Figure 3A) [95]. A smooth landscape has a single global fitness peak that is accessible by an adaptive uphill walk from any genetic background [96,97]; in contrast, a rugged one has multiple local optima [10,96,98], so which peak a population reaches depends on where it starts (i.e., historical contingency) [56,99]. Ruggedness therefore defines how accessible adaptive walks are, and with it the navigability, evolvability and robustness of the graph [55]. A range of measures have been used to quantify ruggedness: the number and density of local optima [96,100]; the fraction of selectively accessible, monotonic uphill mutational paths [8,9,99]; the prevalence of sign and reciprocal-sign epistasis, which is what permits multiple peaks [99,101]; and the autocorrelation, or correlation length, of fitness along a random walk, i.e., how fast fitness decorrelates with mutational distance [102–104].

A more recent set of methods uses spectral graph theory, which describes the shape of a landscape (genotype network) through a spectrum of fitness changes. These graph-based measures include techniques such as determining the Dirichlet energy of the fitness function on the graph to interpret landscape topology, with high Dirichlet energy indicating rapid fitness variation between adjacent sequences, consistent with ruggedness [105–107]. Spectral landscape theory utilises the GFT, which in turn treats fitness as a signal on the genotype graph and decomposes it, separating smooth variation, i.e., fitness that changes gradually between neighbouring sequences, from rugged variation that changes sharply; the standard ruggedness statistics can each be recovered as a summary of the resulting spectrum (Figure 3B) [108–110]. On a complete combinatorial dataset, such decomposition reproduces the additive, pairwise, and higher-order epistasis terms and can also be computed on the sparsely sampled landscapes produced by deep mutational scanning [108].

Much of the theory on quantitative fitness landscapes and their exploration is derived from theoretical models that can be used to represent sequence space, fitness, and the connectivity therein [102,110–112]. These include dense Hamming graphs (nodes as sequences; edges as single-mutation neighbours) such as the elementary landscape [104,105], RMF [113], eggbox [65,114], and NK landscape models [95,111] (Figure 3C). The NK landscape, and its many derivatives [115–118], is the most widely used. Here, *N* is the length of the sequence and *K* is the number of sites each site depends on. Each site is assigned a random fitness contribution that depends on its own state and that of the *K* other residues, with a sequence’s fitness being the average of all *N* contributions. Computing this for all possible sequences gives the landscape of tunable epistasis. At *K* = 0 it gives a smooth, additive landscape, and larger *K* values make it more rugged. Brookes et al. [117], for example, demonstrated that generalised NK models have similar graph Fourier spectra to experimental genotype graphs, establishing that a well-calibrated model can mimic the epistasis of biological systems.

### Sequence similarity networks and hierarchical exploration of protein space

**Figure 4:**
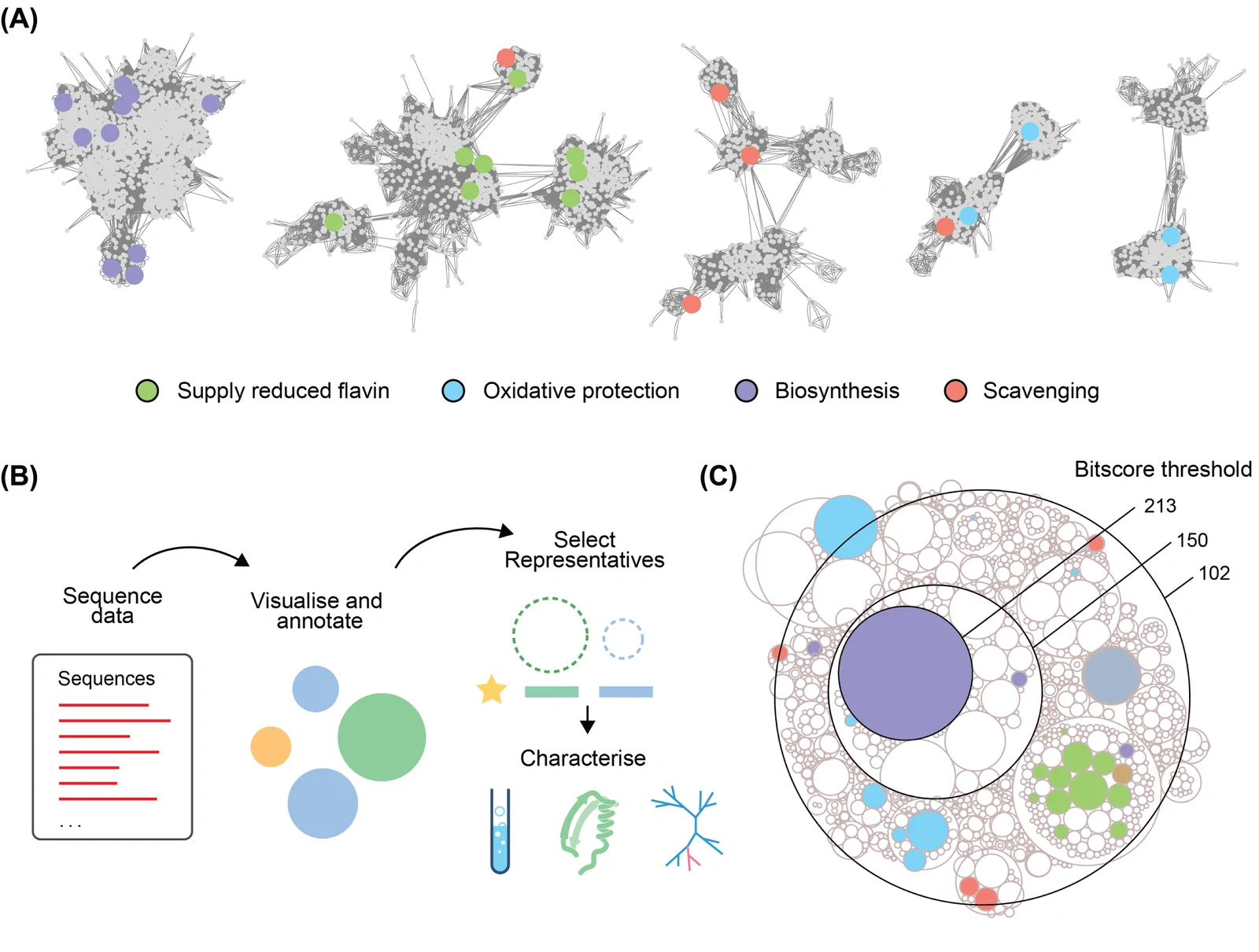
SSNs as tools for exploring sequence space (**A)** Example SSN of a reduced subset of the FMN-binding split barrel superfamily, highlighting clusters with examples of the most prevalent functions. Nodes represent unique sequences, and edges connect sequences if they are above a bitscore threshold of 150. Sequences with known functions are coloured and enlarged. (**B**) Schematic depicting the typical workflow for SSNs, from raw data to visualisation, representative selection, and characterisation. (**C**) An SSN of the FMN-binding split barrel superfamily when visualised using a hierarchical cluster plot. Each circle is an isolated cluster in the SSN at the indicated bitscore threshold, with area scaled by cluster size. Known major functions are coloured with shared colours as panel (A).

With advances in sequencing technology, the number of available protein sequences has greatly expanded and continues to grow. This new wealth of data presents new opportunities for exploring the organisation and functional diversity encoded in natural sequence space, but the scale of the data is incompatible with methods like MSAs. Similarly, over long timescales, evolution cannot be modelled as an adaptive walk on a dense Hamming genotype graph. To explore the sequence space of entire protein superfamilies, the SSN was developed [3]. In an SSN, nodes represent individual protein sequences, and edges connect pairs whose pairwise similarity passes a defined threshold, usually expressed as an *E*-value cutoff [3,12]. By applying force-directed layouts [37], which pull connected nodes together and push unconnected ones apart, sequence neighbourhoods emerge as visually distinct clusters of closely related proteins in the SSN (Figure 4A). Adjusting the similarity threshold resolves these clusters at different levels of homology, from broad superfamilies at low stringency to iso-functional families at high stringency [3]. An important caveat is that SSNs are undirected graphs based on extant homology and hence do not encode explicit evolutionary information or direction. However, the connectivity between distant homologs can be used as a starting point for interrogating potential evolutionary relationships, and SSNs are a useful counterpart to traditional phylogenetic analysis [30].

The value of SSNs lies in their ability to provide a global view of the target sequence space, revealing clustering of similar sequences and connectivity between sequences, which when enriched with functional or taxonomic annotations, can facilitate different analyses and experimental characterisation (Figure 4B). A common application of SSNs is to explore a protein family or superfamily in full, identifying iso-functional clusters and understanding the relationships between subfamilies [3,11,12,119–125]. Once annotated, it can reveal sequence-structure-function relationships, highlighting differences in active-site geometry [123,125], co-factor specificity [12,121], and loop architecture [11,122,125] between subfamilies. Features of the network identified in this way are hypotheses and can be tested directly by characterising representative sequences, for example, by assaying substrate and co-factor specificity [121,123,126]. The same comparison can be made across families: graph alignment of paired SSNs has been used to predict interaction partners between protein families [127]. Connectivity in the network also points to evolutionary relationships between functions. For example, a central “hub” connecting different functions [122] or “linker” sequences that may hint at a path for transition between different functions [123]. These candidates can then be tested more rigorously, using the SSN to choose representatives for phylogenetic inference [12,122,126,128] and reconstructing ancestral sequences along the proposed transitions [126,128]. Finally, an annotated SSN can also be used to specifically explore unknown sequence space. By characterising sequences from clusters without previous annotation, unknown regions can be probed for potential enzymatic activity [129], ligand binding [120,130], or structure determination [119–121]. For example, a recent study systematically catalogued areas of unknown “dark matter” across the UniRef50 database, identifying unclassified protein families [131].

To construct an SSN, the user can perform all-versus-all pairwise alignments (e.g., using BLAST [16], and MMSeqs2 [35]) on their sequence data and then provide the pairwise similarity table to a graph visualisation program such as Cytoscape [3,37]. In addition, the EFI-EST suite of web tools provides convenient ways to construct SSNs, where a user can submit the database identifier (e.g., Pfam and InterPro) for a protein superfamily and the EFI-EST tool will collect the sequence data, construct the SSN, and deliver an annotated Cytoscape file ready for viewing [132]. However, the classic method of directly visualising SSNs has limited scaling, where networks with more than a few million edges rapidly become computationally intractable to visualise. An alternative method (Protein Cluster Tools; PCT) bypasses the scaling problem by omitting edges and instead depicting the SSN as discrete, circular clusters in a nested hierarchy across multiple similarity thresholds [12] (Figure 4C); displaying multiple thresholds also helps in identifying isofunctional clusters, which may not emerge at the same threshold across different functions. PCT can also generate sequence space clusters using pLM-based vector representations, which have been shown to produce comparable clustering to the traditional similarity-based approach with the advantage of being an alignment-free way to compare full-length sequences.

## Structural and interaction graphs

The transition from sequence to structure is a transition from a linear string to a three-dimensional contact network. Cartesian coordinates are one description of a fold. However, the defining feature of a fold is arguably the pattern of local and long-range contacts between residues, not their absolute position in space. While reducing the protein to a graph discards atomistic and dynamic detail, these critical architectural relationships that define the fold are emphasised. The RIN captures these patterns [5]. Nodes represent amino acids, typically Cα or Cβ atoms, and edges represent physical interactions: hydrogen bonds, salt bridges, hydrophobic contacts, or spatial proximity within a cutoff of 5–8 Å. The cutoff is a modelling choice rather than a property of the protein, and conclusions drawn from RINs are tested across reasonable cutoff ranges. The graph inherits the structure behind it, with an edge being either present or absent. A contact from a low-confidence region of a predicted model is indistinguishable from a well-resolved one, and a disordered region missing from an experimental structure appears as absent rather than unknown. The choice of structure, like the choice of cutoff, shapes what an RIN can support.

**Figure 5:**
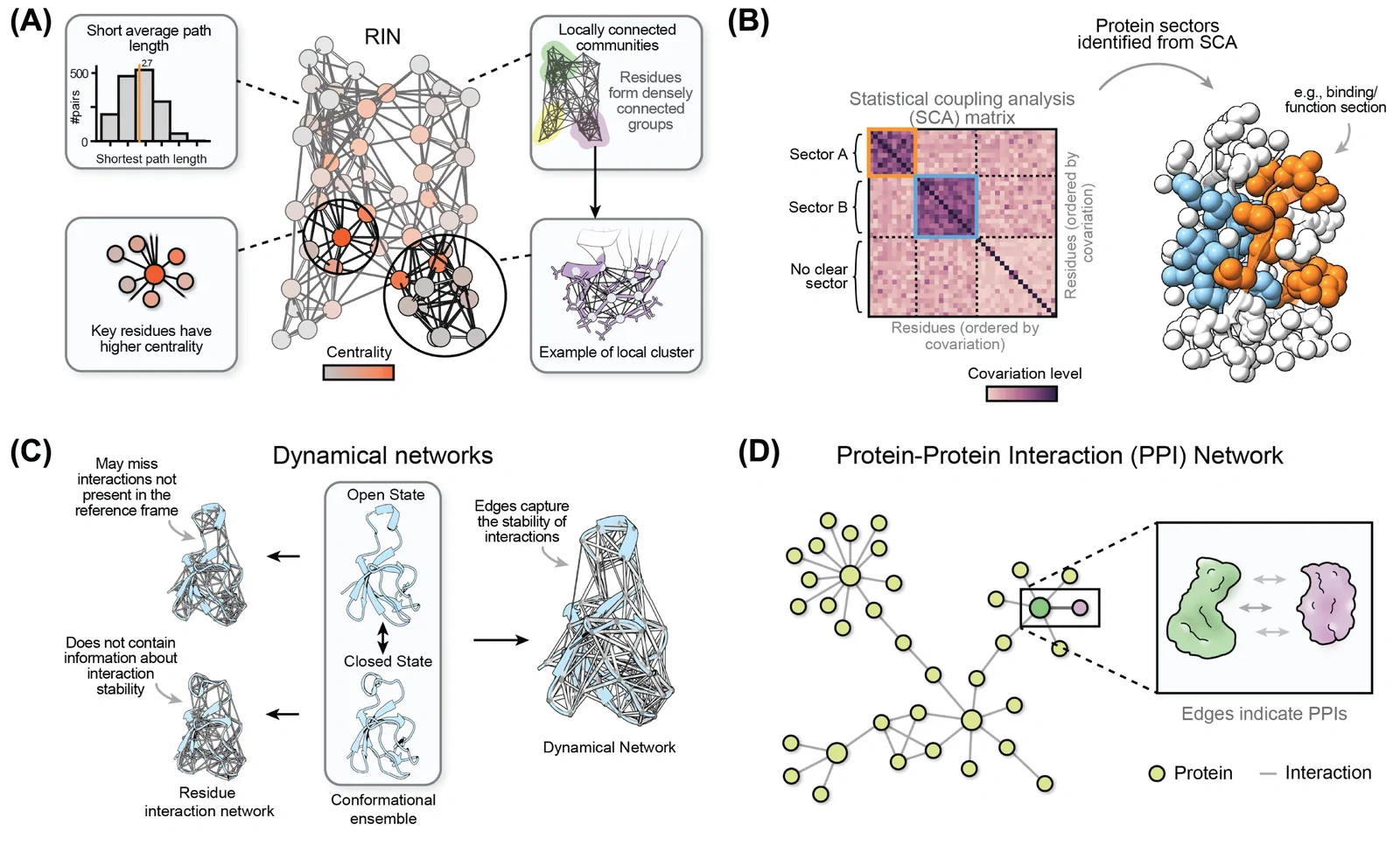
Physical contact graphs (**A**) Interactions in RINs of three-dimensional proteins show small-world network properties, where nodes (residues) are highly clustered and the path length between nodes is on average short. When considering node degree (the number of contacts/interactions) for each residue, high centrality (high connectivity) is associated with key residues. (**B**) Statistical coupling analysis (SCA) quantifies the covariation between every pair of sites in an MSA, producing a matrix that can be read as a weighted graph in which all residues are connected, and edge weights are covariation scores. Densely covarying groups define sectors, sets of residues that covary more with each other than with the rest of the protein. Sectors are usually contiguous in the folded structure and can sometimes be separated into groups associated with different properties. (**C**) A conceptual example of a dynamical network that is built using information from protein dynamics rather than a stationary structure to generate a contact graph. (**D**) A PPI network where nodes indicate proteins and edges connect proteins that interact with one another.

A central insight from this topological perspective was the discovery that proteins are small-world networks [1,133] (Figure 5A). Atilgan et al. [1] applied the Watts-Strogatz framework [134] to protein structures and observed that residue networks possess a dual nature: high local clustering (residues interact with sequential neighbours and within local secondary structure) combined with short average path lengths between any two nodes. This is achieved through long-range contacts, edges connecting residues far apart in sequence but adjacent in the folded structure. This topology is likely to be functionally important. A protein must be stable enough to maintain its fold (high clustering) yet dynamic enough to transmit signals (short path lengths). The graph topology provides a physical mechanism for allostery: signals can propagate along specific, evolutionarily conserved pathways defined by network topology. Residues with high betweenness centrality, i.e., nodes lying on the shortest paths between many pairs of other nodes, act as information bottlenecks and are statistically enriched in active sites and allosteric hinges [135,136] (Figure 5A). A worked example is provided by PDZ domains, where statistical coupling and network analyses identified residues coupling the ligand-binding pocket to distal sites, in agreement with experimental allosteric perturbation studies [137–139].

SCA, developed by Lockless and Ranganathan [137] and extended into protein sectors by Halabi et al. [140], uses correlated substitutions across an MSA to identify networks of co-evolving residues. The SCA matrix is the weighted adjacency matrix (a table of edge weights for all pairs of nodes/residues) of a graph in which positions are nodes and edges encode statistical coupling, and a sector is a community in this graph, i.e., a set of positions coupled densely to one another and weakly to the rest (Figure 5B). Halabi et al. found sectors to be sparse, spatially contiguous in the fold, and sometimes separable into several that each track different properties such as ligand specificity, allosteric communication, or thermal stability. This correspondence is only partial: much of an SCA matrix’s weight comes from single-site conservation rather than coupling, so a single sector within a protein may be a cluster of separately conserved residues [141]. Additionally, the long-range coevolutionary signal that sectors are built from appears to be mostly indirect correlation propagated through contacting residues, with the direct couplings being mostly physical contacts [142].

An RIN is derived from a single structure, yet the fold it represents is not static, and some allostery operates through changes in the amplitude and correlation of thermal fluctuations rather than any shift in mean structure [143]. Dynamical network analysis captures this while keeping the graph a physical contact graph: the edges are still residue contacts, but weighted by their persistence and correlated motion across a molecular dynamics ensemble, so centrality and community structure now reflect how the fold moves rather than how a single conformer sits [144] (Figure 5C). Here, the graph is only as good as the ensemble; correlation measures and contact-persistence thresholds matter as much as the distance cutoff for the static counterpart. The information added with dynamics carries an evolutionary signal of its own: in influenza NS1, mutations far from the binding interface raise ligand affinity by rewiring the dynamic network, and the highest-betweenness residues that carry communication between communities are conserved across viral evolution [145]. Epistasis here is a property of the network rather than of any single contact; a substitution redistributes centrality across the ensemble, and the contacts that evolution conserves those holding the communication paths together.

At the cellular level the same graph description applies. In a PPI network, proteins are nodes and physical binding events are edges (Figure 5D). A few proteins have many interaction partners, while most have only a handful. This pattern is often described as scale-free, meaning the number of partners follows a power law dominated by a small number of highly connected hubs [146]. However, large surveys find strictly scale-free structure to be rare, with log-normal distributions fitting most degree data as well or better [147], and much of the apparent power law in PPI data reflects uneven study effort across proteins rather than interactome biology [148]. Two properties nonetheless follow this skewed distribution: random protein loss rarely disrupts global connectivity, whereas targeted hub removal is disproportionately disruptive [149], and high-degree proteins are statistically more likely to be essential [], though this centrality-lethality correlation is partly inflated by study bias [148] and is better explained by membership in essential complexes than by hub status itself [150]. PPI networks are also organised into modules corresponding to functional complexes such as the ribosome or proteasome [151], recoverable by graph-clustering algorithms without prior biological knowledge [152]. The practical biochemical consequence is that druggability and functional impact depend on topological position in the cellular graph as well as on local chemistry, and a single substitution within an RIN can propagate outward to change PPI edges [153,154].

## Geometric deep learning and graph-centric ML

**Figure 6:**
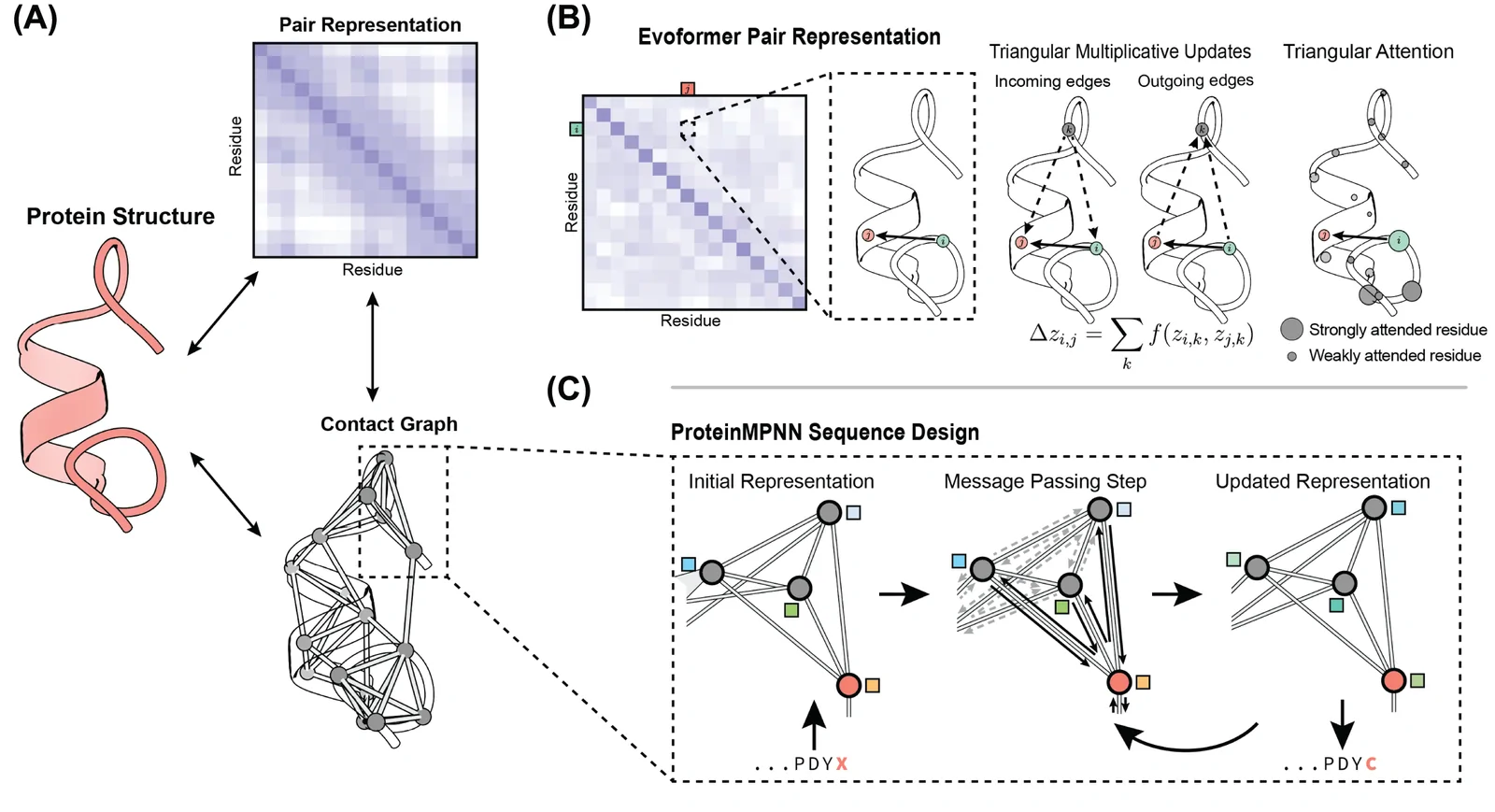
Geometric deep learning for protein structure and design (**A**) Proteins are three-dimensional structures in Euclidean space, but their structure and function are relational, so contact graphs and pair representations are a convenient way to capture topology along with the distances and orientations that stay fixed under rotation and translation. (**B**) A graph-theoretic interpretation of AlphaFold2's pair representations. The Evoformer uses an internal pair representation to capture evolutionary and physical interactions between residues, which can be interpreted as edge features on a dense residue-residue graph. Triangular updates refine the features of each residue pair using the two other sides of every triangle it forms with a third residue, pushing the pair features towards geometric consistency. (**C**) ProteinMPNN operates over the sparse contact graph of a protein. Message passing over this graph produces context-dependent residue embeddings that encode the local structural environment and are used to predict the conditional probability of each amino acid identity at a given position.

The capacity of graphs to capture the intricacies of protein structure, function, sequence, and evolution is well established in the literature and continues to be developed and expanded. With the rapid uptake of machine learning approaches in the biological sciences, geometric deep learning, a framework designed to process data with complex, non-Euclidean structure, such as graphs, manifolds, and 3D shapes, has seen its utility and success in biology become increasingly apparent. Geometric deep learning in protein science seeks to build models and training paradigms that effectively infer and exploit this relational data structure, including its topology and symmetries [155]. Consequently, geometric deep learning has become an important approach in biological machine learning, underpinning many modern methods for protein structure prediction, protein design, and functional prediction (Figure 6A).

Modern protein structure prediction models exemplify the central principle of geometric deep learning. Graphs are well-suited to capturing/representing the rotational and translational symmetry of protein structure. A structure is unchanged by rigid-body rotation and translation, so the quantities that define it are the relative distances and orientations between residues rather than their absolute coordinates [156]. Graphs capture this directly, though other approaches can also capture structures independently of the coordinate frame. Shape-mers, descriptors that group structural fragments by their rotation-invariant moments, can describe a protein as a vector of fixed length [157]. Structure prediction models also take a relational route. Rather than reasoning solely over linear amino acid sequences, models such as AlphaFold2, AlphaFold3, and RoseTTAFold maintain pairwise representations that encode relationships between residues and enable information to propagate through the network of interactions that defines a protein structure [17,158,159]. Although AlphaFold2 and AlphaFold3 are transformer-based architectures that do not internally represent these residue interactions as graphs, they can be viewed as implicitly operating over the pairwise graph of residues to reason about protein structure. Consistent with this interpretation, the Evoformer is described as a solution to protein structure prediction as a graph inference problem in 3D space [17].

In AlphaFold2 the pair representation stores, for every residue pair (*i*,*j*), learned features describing their relationship, which the Evoformer refines using evolutionary signal drawn from the MSA [17]. The key operation is the triangular update, in which the features of a pair (*i*,*j*) are updated using the two other sides (*i*,*k*) and (*k*,*j*) of each triangle it forms with a third residue k, pushing the learned relationships towards geometric consistency [17] (Figure 6B). AlphaFold3 generalises this from residues to the components of an arbitrary molecular complex, including ligands and nucleic acids, so the pair representation now spans the whole chemical system [158]. It carries no persistent MSA through the main processing stack; evolutionary information is compressed early into the pairwise features, which a Pairformer refines, and a diffusion module turns into coordinates [158]. ESMFold removes the alignment, deriving the pairwise representation from the ESM protein language model rather than an MSA [160]. The pair representation remains the object being inferred; however, with ESMFold, evolutionary weights are drawn from the language model rather than an alignment.

The RoseTTAFold family of models extends this idea by explicitly representing proteins and ligands as graphs within its 3-track architecture, which includes representations for the sequence, residue pairs, and three-dimensional coordinates, passing information between all three tracks at each block for structure prediction and refinement [159,161,162]. The 3-track block builds in a further geometric inductive bias. Inter-residue distances and orientations are invariant to rigid-body motion, but a model that outputs coordinates must be equivariant, with its prediction rotating and translating with the input. RoseTTAFold provides this through transformers equivariant to SE(3), the group of rigid-body motions that comprises all rotations and translations [156], letting it reason about protein geometry independently of the coordinate frame. Together, these models demonstrate that geometric reasoning can emerge from both dense pairwise representations and explicit GNN architectures, provided the underlying relational structure of proteins is incorporated as an inductive bias.

Protein sequence design can be viewed as the inverse of protein structure prediction: rather than predicting structure from sequence, it seeks to identify sequences that are compatible with a specified structure [163]. The identity of a given position will often be most influenced by its surrounding environment, with the positions of its neighbouring residues dictating features like solvent accessibility, possible interaction partners, and steric constraints [33,164]. As such, sequence design can naturally be formulated as a constraint-satisfaction problem on a residue-contact graph, where the goal is to assign an amino acid identity to each residue given the known inter-residue contacts and distances. ProteinMPNN addresses sequence design from this perspective by constructing a sparse graph in which each residue is connected to its *k* nearest neighbours. Nodes represent residues, and edges encode geometric information, including inter-residue distances and relative orientations derived from the backbone structure [33]. Restricting edges to be between physically close nodes encourages the model to reason over the local context of each residue, introducing an inductive bias that mirrors the physical interactions governing protein stability and folding. Message passing over this graph produces contextual residue representations that encode the local structural environment, which are then used by an autoregressive decoder to predict amino acid identities conditioned on both the backbone structure and previously assigned residues. By using these structure-informed representations, ProteinMPNN captures dependencies between interacting residues and generates sequences that are compatible with a specified fold. The same approach extends to other constraints: LigandMPNN adds a ligand-residue graph, connecting each residue to nearby ligand atoms so design can be conditioned on bound small molecules [165], and SolMPNN biases design towards soluble sequences [166]. The success of ProteinMPNN [33], LigandMPNN [165], and SolMPNN [166] demonstrates how graph representations can encode biologically meaningful inductive biases, enabling sequence design models to reason directly over the structural environments that govern protein stability and function.

The same geometric machinery also drives generative design. RFdiffusion fine-tunes the RoseTTAFold structure network into a diffusion model, denoising random coordinates into novel backbones while respecting the SE(3) symmetry of the fold; the generated backbones are then sequenced by a model such as ProteinMPNN [51]. Structure prediction, generation, and sequence design therefore share one representation: a protein as a graph of geometric relationships between residues.

The close relationship between structure and function has also motivated the integration of structural graphs into models designed for function prediction and annotation. LM-GVP (a protein language model combined with a geometric vector perceptron graph network) combines a protein language model with a structure-based GNN, resulting in better property prediction and function annotation [27]. Structure-informed language models instead incorporate backbone coordinates directly into sequence representations and have been used to evolve protein and antibody complexes without supervision [28]. Joint sequence–structure embeddings go further, learning a shared representation from MSAs and structural graphs that modestly outperforms MSA-only and structure-only baselines on variant-effect prediction tasks, with the gain most evident in proteins with limited homologues [29]. The point is not that graphs are replacing alignments. Rather, some of the most effective representations integrate evolutionary information from sequences and MSAs with geometric and relational constraints encoded in structural and interaction graphs [167,168].

The utility of graph-based representations in protein machine learning extends beyond residue-interaction networks. Proteins, ligands, nucleic acids and other biomolecules can all be represented as graphs, allowing models to encode both covalent connectivity and non-covalent interactions. This principle underlies many contemporary biomolecular modelling approaches. Machine learning can also be used to make fitness landscapes more navigable, such as by learning smooth representations of rugged fitness landscapes or by constraining models to better capture the underlying features of the biological fitness landscape [169,170].

### Knowledge graphs

**Figure 7:**
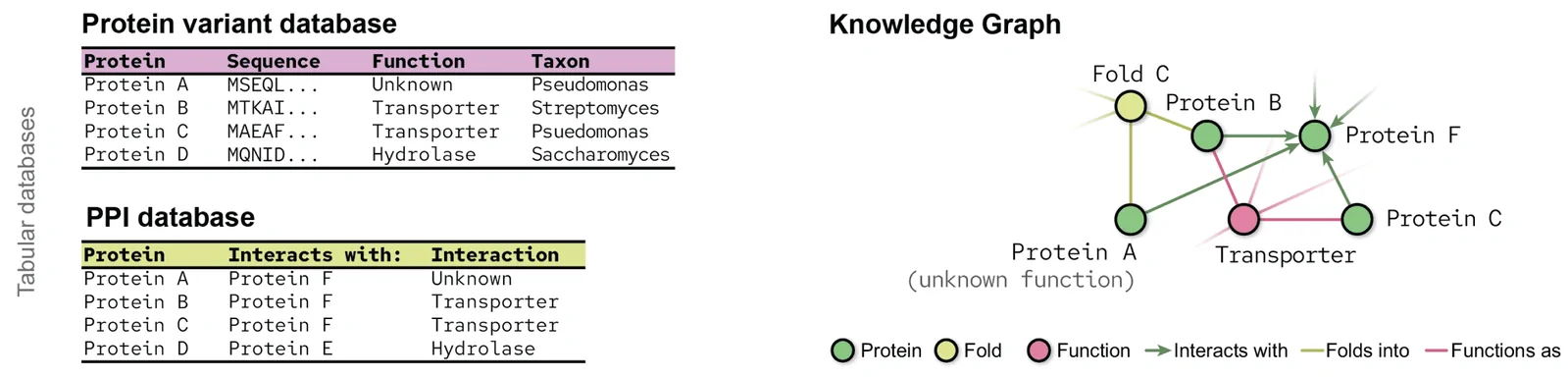
Knowledge graphs for proteins The transition from a flat database to a knowledge graph. **Left:** protein entries as rows in tabulated data. **Right:** A simple knowledge graph containing multiple protein properties.

Most members of large enzyme superfamilies have no experimental annotation, and for many superfamilies the characterised fraction is only a few percent [171]; homology transfer fills some of this gap, but it fails exactly where it is most needed, between sequences too divergent to align reliably. Function in these cases must be inferred from evidence that the sequence alone does not carry: the genomic neighbourhood a gene sits in, the structural fold it adopts, and the reactions its relatives catalyse. A knowledge graph is the data structure that holds these heterogeneous relationships together. Where a database stores entries as independent rows, a knowledge graph stores them as typed edges between entities, so that proteins, structures, reactions, genome contexts, and literature become a single connected network in which missing edges can be inferred from the pattern of known ones [172] (Figure 7).

The genomic-context route is already standard in enzymology. EFI-GNT, the companion to the EFI-EST tool used to build SSNs, takes the clusters of an SSN and retrieves the genome neighbourhood of every sequence in each, so that an unannotated cluster can be assigned a likely function from the gene neighbourhoods its members share [132]. This depends on the functionally related genes being physically co-localised, which holds for bacterial operons and metabolic gene clusters of fungi but not for other eukaryotic genes [132]. The same logic generalises across evidence types: if an uncharacterised protein A sits in the same operon as a known dehydrogenase and shares a fold with a known reductase; the graph supports a functional hypothesis for A with no detectable sequence identity to either. Larger biological knowledge graphs apply this at scale, integrating interaction, ontology, phenotype, and literature databases and learning to predict missing edges, which has been used to annotate protein function, interactions, and disease associations [172]. Resources such as STRING [43], Reactome [46], the Gene Ontology and GO-CAM [47], and UniProt’s linked-data infrastructure [171] provide much of the underlying connected data.

While knowledge graphs are useful, there are some risks. Predicting a missing edge assumes that two entities connected to many of the same things are probably connected to each other too, sometimes referred to as ‘guilt by association’ [173,174]. When a graph is built largely from computationally inferred annotations, inference over it risks circular evidence, predicted edges resting on edges that were themselves predicted. Reliability is also uneven: in gene-association networks, functional signal concentrates in a few informative connections, so benchmark performance overstates the accuracy of new predictions [175], and whether this holds for heterogeneous, multi-relational graphs is untested.

### Limitations of graphs

It is important to note that graphs are abstractions, and the choices made in their construction constrain their utility. We highlight some of the most important caveats here before discussing future directions.

The first caveats concern construction and data. Graph construction is a modelling choice rather than a discovery: for example, the cutoff used to define an RIN edge (5 Å versus 8 Å, Cα versus Cβ, with or without sequence-adjacent contacts), the bitscore threshold of an SSN, and the confidence cutoff applied to a PPI network all change the resulting topology and any centrality, community, or spectral metric computed from it. There is no canonical, parameter-free graph for any of these systems, and conclusions should be tested for robustness across reasonable construction choices, e.g., a range of bitscore thresholds in SSNs. The data underlying biological graphs are also noisy and incomplete: PPI networks contain false positives from non-physiological interactions and false negatives from undetected ones, biases from differential study of well-known proteins, and edge weights with often unquantified uncertainty. Strong topological conclusions drawn from a single dataset are correspondingly risky. The second group of caveats concerns dynamics and learning. Proteins are not static graphs: functional dynamics, allosteric communication, intrinsic disorder, and induced-fit binding involve time-dependent sampling of different substates. Dynamical network analysis recovers part of this, as discussed above, but most graphs in practice are still read from a single structure, often a computational prediction that carries its own error. Treating such a snapshot as ground truth obscures the dynamics it averages over, and for predicted structures, does so over a state that was never physically sampled. Thirdly, graph-learning methods inherit biases from their training data and from the construction step itself: GNNs trained mostly on well-studied folds, soluble proteins, or particular interaction types need not generalise, and over-smoothing and the brittleness of message passing on noisy graphs remain open problems. Graph representations are therefore a complement to, not a replacement for, biochemical and biophysical reasoning. Used carefully, they augment our intuition, generate hypotheses, and yield quantitative metrics difficult to obtain from sequence or structure alone; used uncritically, they produce confident-sounding numbers from arbitrary modelling choices. The questions worth asking of any graph-based result are the same as one would ask of any biochemical result: what was assumed, how robust is the conclusion, and, most importantly, what experiment would falsify it?

## Future directions: graph representations for protein data at scale

Data growth is itself a driver of graph methods. As sequence and structure databases expand, the most immediate problem is search: the performance of many computational methods, such as structure prediction and training deep neural networks, scales with the diversity and number of available sequences [17,19,176,177], and finding the relevant sequences in ever-larger collections becomes harder [35,178]. One approach is to represent sequence databases as annotated k-mer-based graphs. Using this strategy, Karasikov et al. [179] demonstrated faster search performance and smaller index sizes than conventional indexing methods while maintaining sensitivity.

A related shift is from searching sequences to searching structures. Graph alignment may increasingly augment sequence alignment for homology search: rather than comparing strings of letters, RINs themselves can be compared. Graph isomorphism, determining if two graphs have the same connective pattern, is computationally demanding, but graph embedding offers a scalable approximation: by mapping every protein graph into a continuous vector space, homology search becomes a nearest-neighbour problem in this learned space. Building on general-purpose graph embedding methods [180], protein-specific approaches are now practical. Greener and Jamali developed Progres [181], in which protein domains are represented as structure graphs and mapped by a GNN with supervised contrastive learning into low-dimensional embeddings, enabling rapid nearest-neighbour structure search across large predicted-structure resources. Related tools such as Foldseek [182] use compact structural alphabets to make all-versus-all structural search tractable at the scale of the AlphaFold Database. In such systems, two proteins may be close not because they share obvious sequence similarity, but because their structural graphs encode similar topological features, an analogue of sequence search at the level of structural topology.

More data should also refine concepts that are currently data limited. Statistical coupling and sector analysis depend on deep, well-sampled alignments; as superfamilies fill in, it should become possible to test more directly whether sectors decompose into separable functional units, a question present data leave unsettled. Denser genotype sampling will similarly extend fitness-landscape analyses from single proteins towards whole families.

A further direction is integration across data types. Integrating multi-omics may begin to break down the boundaries between protein science and systems biology: in principle a protein node can be connected to the metabolites it modifies, the regulatory RNAs that act on it, and the cellular phenotypes it influences. The same multi-relational structure is what lets learning models combine data types: the joint sequence-structure models discussed earlier already use two modalities, and the natural extension is to add interaction, pathway, and phenotype edges so that one model reasons across several scales at once. Whether such pan-omic graphs deliver useful integration in practice will depend on annotation quality and on continued biochemical scrutiny of the inferences they support.

Finally, directionality in graphs is likely to become an increasingly studied aspect. Most graphs in the present review are undirected, recording which entities relate but not which way evolution or causation flows; directed graphs encode that asymmetry, and evolutionary trajectories are a natural target, though this is still an emerging use. Phylogenetic trees are already directed graphs, used to infer ancestral sequences over a bifurcating topology that assumes site independence [183]. More general directed graphs relax these assumptions. A variant evolution graph built over dated SARS-CoV-2 genomes captures the recombination and convergence that trees miss [184], and protein language models have been used to orient the edges of an SSN by increasing sequence pseudo-likelihood, often recovering known directionality [185]. A related goal in protein engineering is to predict the path from a suboptimal sequence to a fitness optimum, the sink node of such a graph from which no edges lead uphill further [186]. Activity here is still limited, but because evolution over sequence space is directional [187], constructing and interpreting these graphs is a plausible direction for future work.

## Conclusion

Because the sequence remains the most compact and experimentally accessible representation of a protein, much of protein science has been built on the linear sequence and its alignments. The present review does not suggest that this coordinate system should be replaced, but that many current problems are explicitly relational and better posed on a graph (Table 2). Protein structures, evolutionary landscapes, and interactomes are systems of relationships, and graph-theoretic representations make those relationships the primary object of analysis. We have followed this perspective from the theory of sequence space and fitness landscapes, through the exploration tools of SSNs and hierarchical graphs, to the geometric representations used in AlphaFold, ProteinMPNN, and RFdiffusion. Many of the most successful predictive models in modern protein science now incorporate graph-like or geometric representations. As the field confronts remote homology, epistatic landscapes, and *de novo* design, the quantitative metrics of graph theory, including spectral gaps, centrality, connectivity, and graph expansion, are likely to become a routine part of the protein-analysis toolkit, augmenting rather than replacing the substitution-matrix-based logic of classical sequence analysis. Increasingly, graph-based methods represent powerful and complementary approaches to both classical bioinformatics and wet-lab approaches, helping to resolve the underlying complexity of biology across scales.

**Table 2:** Which graph should I use?

Biological problem	Recommended graph approach
Mapping sequence-function space across thousands of related enzyme sequences.	SSN with hierarchical clustering (EFI-EST, ProteinClusterTools).
Identifying candidate allosteric or catalytic hot spots from a protein structure.	RIN with centrality and community analysis (RINalyzer, NetworkX).
Characterising a fitness landscape from DMS data.	Genotype graph with accessibility, ruggedness and spectral analyses.
Searching a large library of predicted structures for homologues of a query.	Graph embedding plus nearest-neighbour structure search (Progres [181], Foldseek [182]).
Inferring function for unannotated metagenomic proteins.	SSN combined with genomic-neighbourhood analysis and a knowledge graph (EFI-EST [132], STRING [43], UniProt RDF [171]).
Solving an inverse design problem with a fixed backbone.	Message-passing GNN on the structural graph (ProteinMPNN [33]).
Predicting ligand poses given a binding pocket.	Geometric or diffusion model on a combined protein-ligand graph.

Different biological questions are best matched to different graph representations. The pairings below summarise the most common combinations encountered in protein science.

## Open Access

Open access for this article was enabled by the participation of the Australian National University in an all-inclusive Read & Publish agreement with Portland Press and the Biochemical Society under an agreement with CAUL.

## Abbreviations

BLAST: basic local alignment search tool
GFT: graph Fourier transform
GNN: graph neural network
MSA: multiple sequence alignment
PPI: protein–protein interaction
RIN: residue interaction network
RMF: rough Mount Fuji
SCA: statistical coupling analysis
SSN: sequence similarity network
